# Light Assessment Pathway Creation and Implementation in the North London NHS Foundation Trust (NLFT) Perinatal Service

**DOI:** 10.1192/bjo.2026.11426

**Published:** 2026-06-30

**Authors:** Stavroula Madenlidou, Naashoma Carvalho, Pia Ghosh

**Affiliations:** North London NHS Foundation Trust, London, United Kingdom

## Abstract

**Aims:**

The 10% access rate target for perinatal mental health services in the UK was introduced as part of NHS England’s broader strategy to expand and improve access to specialist perinatal mental health care. Within our organisation, the 10% target was actively discussed and various strategies had been implemented to achieve the target. It became evident that women with mental health difficulties on the milder spectrum, where primary care interventions were appropriate, a full new patient assessment (NPA) was not indicated. The hope was that a light assessment (LA) pathway would develop a more responsive pathway for referrals which would be more suitable for other services, it would allow signposting for mild and less complex referrals to the appropriate services in a more timely manner and it would allow clinicians to prioritise time on more complex presentations.

**Methods:**

The aim was to implement a LA pathway to support the service to reach the 10% access target.

Consultation with other perinatal teams around the country followed. Concerns on balancing seeing milder cases while preventing team burnout were also shared by them and options for a less in-depth initial assessment in these cases were explored.

The NLFT service agreed to a trial of a LA pathway for mild cases. A LA template and guidance on its use, were created and subsequently discussed in service-wide meetings and adjusted according to feedback. It was formally implemented on 27 January 2025 with plan for feedback meeting in 4 weeks and total trial of 6 months.

The NLFT perinatal service, consists of 3 teams. Two different systems were implemented based on the preference of the individual teams, differing on the days/hours duty service isrun and the days LA appointments are scheduled. In both systems LAs could be converted to NPAs if it becomes evident during the appointment that the patient requires a more in-depth assessment.

**Results:**

For the period of the LA pilot from 27 January 2025 to 30 May 2025, the specialist perinatal service received 684 referrals. All referrals were accepted, unless they were out of borough or not within the perinatal period.

Of the 684 referrals received, 71 (10%) were allocated for a light assessment. 81% of the women allocated for light assessments attended their assessments. 15% (11) allocated for a light assessment, did not attend their assessment and were discharged and 2.5% (2) declined the assessment. Of the 2 that declined the assessment, one was discharged and 1 requested for an NPA, assessed and subsequently discharged. Of the 58 women who attended the LA, 74% (43) were assessed and discharged and 21% (15) were assessed and taken on. The data across the 3 teams were very similar, other than the number of days from triage to assessment. In the south team, the assessments were scheduled within 5 weeks from referral whereas in the west and east teams all assessments were scheduled within 3 weeks from referral.

Qualitative staff feedback was gathered via questionnaires. The main points brought up are as follows:

- Better triaging guidance needed for newer staff.
- More information needed on risks and more info in referrals.
- If uncertain based on info on the referral form, request for more info, if still not sufficient refer for a full NPA.
- LA’s are helpful for efficient signposting and time-saving. They work well when patients are appropriately triaged. Women are seen, sign posted and discharged in a week.
- Seeing moderate to severe people quicker. People moved through the service quicker, reducing caseloads.
- LAx template very clear.

LA are held by Duty, rather than individual clinicians. Better to be a little bit stressed on Duty for longer term gain.

Patient questionnaires were created and were sent out after the LA was conducted.

**Conclusion:**

Setting up the LA pathway has allowed the team to broaden the number of women they see, while preventing team burnout and achieving better resource allocation to SMIs (serious mental illness).

Some of the challenges faced during this project include:

- Unexpected loss of trust video-call system.
- Some staff anxiety whether any important information was missed (this is managed by presenting cases at the weekly MDT).
- Some of the LAs being re-referred (this is monitored and re-referrals are allocated an NPA).
- Limited information on referrals.
- Poor response rate on patient feedback questionnaires.

Other reflections and considerations:

- Based on feedback on how the name “Light Assessment” can be perceived amongst other teams and patients, the service changed the name “Light Assessment” so as not to diminish the work being done.
- Request readjusting the 10% target for the reduced birth rate in the area.
- From 21/1/25 self-referral links were also implemented which improved the access rate to the service further.

Next steps:

The feedback overwhelmingly directed towards continuing with the light assessment pathway.

Improve the triage process to the appropriate assessment pathway and consideration of conducting education to referrers of the information needed. Following discussions, it was agreed that when receiving an inappropriate referral we would seek further information from the referrer before accepting it. The team continues to conduct teaching sessions to other teams and this will be highlighted during teaching.

Some NLFT perinatal teams experienced difficulties with primary care teams accepting the referrals from our team as their impression had been that once a referral was accepted by our teams for assessment, the referral would be appropriate for secondary care. The change in the way the team operates in accepting and managing referrals will be highlighted to them. Referrers will also be made aware to inform patients of how the team operates in order to manage expectations.

Details of the pilot were shared with other perinatal teams in LSPCA meetings. Other perinatal services have also reached out to our team and details of the light assessment pathway has been shared with them so that it can be implemented elsewhere.

